# The Board of Visitors of the Balto. College of Dental Surgery

**Published:** 1881-02

**Authors:** 


*The Constitution, Powers and Duties of the Board of Visitors of the Baltimore College of Dental Surgery.—*

Art. I.—The Board of Visitors of the Baltimore College of Dental Surgery consists of members appointed or elected by the Faculty of the College at their discretion.

Art. II.—Resignations of Membership in the Board should be presented to the Faculty, who alone have power to fill all vacancies occurring from any cause.

Art. III.—The Officers of the Board shall appoint each year a committee of three visitors, whose special duty it shall be (they consenting) to attend the final examinations of the Graduating Class, and to advise with the Faculty as to the qualifications of the Candidates for the degree of "Doctor of Dental Surgery." It is understood that the final examinations will be held during the week preceding the Commencement week of each year, and the Faculty of the College will endeavor to complete these examinations in four days, allowing three days to intervene between the time of the announcement of the result of the examinations and Commencement day.

Art. IV.—It is the duty of the Board of Visitors, or as many of them as can do so, to attend the Commencement of the College each and every year.

Art. V.—The Board of Visitors shall determine to whom the several Collegiate Prizes shall be awarded; and said Prizes shall be awarded at the Commencement by the President of the Board of Visitors.

Art. VI.—The Officers of the Board shall be a President, a Vice President and a Secretary, who shall perform the duties usually appertaining to their respective offices.

The Officers shall be elected at a regular Annual Meeting, and shall hold their offices for one year or until their successors are elected.

Art. VII.—The regular meeting of the Board shall be held at 2 o'clock P. M. on the day immediately preceding the Commencement in each and every year.

The Board may adjourn from time to time to complete the business of the meeting.

Art. VIII.—The President may call special meetings of the Board at his own discretion, or at the request of the Faculty, or of three members of the Board. The Secretary shall give to each member of the Board due notice of all regular and special meetings.

**Art. IX.—At any meeting of which due notice shall have been given, the Visitors present shall possess all power belonging to the whole Board of Visitors.**

**The Officers of the Board shall prepare the "Visitors' Letter" to accompany and be published in the Annual Catalogue issued by the Faculty, such letter having been first approved by the Faculty.**

---

### BIBLIOGRAPHICAL.

